# Governing Campus Speech: a Bottom-Up Approach

**DOI:** 10.1007/s12115-018-0279-1

**Published:** 2018-08-03

**Authors:** Emily Chamlee-Wright

**Affiliations:** Institute for Humane Studies, George Mason University, Vernon Smith Hall, 3434 Washington Blvd, Arlington, VA 22201 USA

**Keywords:** University governance, Campus speech, Academic freedom, Polycentric order, Emergent order

## Abstract

I review arguments favoring bans on problematic speech (e.g., hate speech) on campus. Contrasting such calls for top-down regulation, I explore the potential for a “bottom-up approach” to campus speech governance to address vexing concerns pertaining to campus speech without violating free speech and academic freedom principles. I examine the political economy and epistemological dynamics inherent within the two forms of governance. I argue that, relative to a centralized top-down approach, a decentralized bottom-up approach to speech governance avoids political dynamics that bend toward the inappropriate use of power. Further, I argue that a bottom-up approach leaves the discursive space contestable, and therefore more open to new discovery and correction. Recognizing that a bottom up approach is no panacea, I also consider open questions and concerns that warrant further inquiry.

## Introduction

Debates about campus speech tend to toggle between full-throated endorsements of First Amendment rights and calls for administrative prohibitions against speech that is clearly false and/or works against the effort to create an inclusive learning environment. Free speech advocates draw upon classic defenses of speech freedoms in the liberal tradition, such as John Stuart Mill’s ([1859] 2002) *On Liberty*, and Alexander Meiklejohn’s ([1948] 2012) *Free Speech and its Relation to Self-Government*. Free speech advocates warn against any effort to regulate speech, arguing that even in egregious cases of false or offensive speech, the dangers of speech regulation outweigh the perceived benefits, particularly in a university setting, where the unfettered exchange of ideas is essential to the mission of higher education. Drawing upon the liberal tradition, scholars such as Nadine Strossen (2018), Lukianoff and Haidt (2018), Donald Downs (2005), Keith Whittington (2018), Jonathan Rauch (2013), and Erwin Chemerinski and Howard Gillman (2017) have defended speech freedoms on the grounds that intellectual and social progress, including and especially progress on issues related to social justice concerns, fundamentally depend on freedoms of speech and expression.

Advocates of university prohibitions on hate speech, on the other hand, draw upon philosophical traditions such as the Frankfurt School, critical legal studies, and critical identities theory. See, for example, the works of Herbert Marcuse (1965), Stanley Fish (1994), Kimberle Crenshaw (1989), Charles Lawrence (1993), Mari Matsuda (1993), and Richard Delgado and Jean Stefancic (2017). Scholars working within these traditions argue that liberal arguments favoring First Amendment rights, at the very least, miss the point of their concerns; at worst, they are perpetuating a system of social dominance and violence designed to keep members of marginalized social groups “in their place.” Critics on this side of the debate argue that the university setting is exactly the wrong place to hold to a hardline free speech position. Allowing Holocaust deniers, white supremacists and other purveyors of false speech a platform on campus runs counter to the truth-seeking mission of higher education. Further, such speech, they argue, constitutes harm in that it inhibits learning, particularly for students in marginalized groups, and should therefore be prohibited in any learning environment.

Below, I review the case favoring top-down rules restricting problematic speech in the academy. I then explore the self-governing properties of speech, and the potential for a “bottom-up approach” to campus speech governance. In doing so, I examine the political economy and epistemological dynamics inherent within the two forms of speech governance. I argue that, relative to a centralized top-down approach, a decentralized bottom-up approach to speech governance avoids political dynamics that bend toward the inappropriate use of power. Further, I argue that a bottom-up approach to speech governance allows individual members of the academic community to make better use of discipline-based expertise and local knowledge cultivated through routine professional practice. Further still, I argue that unlike a top-down approach to speech governance, a bottom-up approach leaves the discursive space contestable, and therefore more open to new discovery and correction. In the penultimate section, I address open questions and concerns that warrant further inquiry. I conclude that a bottom-up approach to speech governance has the potential to address vexing concerns pertaining to campus speech without violating the principles of free speech and academic freedom.

## The Case for Prohibiting Certain Speech within the Academy

In his 1965 essay “Repressive Tolerance,” Marcuse challenges the liberal assumption that speech is benign. He advocates for the withdrawal of toleration of speech and assembly for those engaged in repressive speech, i.e., speech that discriminates against marginalized groups and opposes social welfare policies. Such speech, he argues, reinforces repressive social structures, serves the interests of those who hold power, and continues a pattern of oppression of marginalized groups.

Similarly, Fish (1994), Matsuda (1993), and Waldron (2012) argue for regulatory prohibitions of hate speech—speech that is aimed at demeaning and/or intimidating marginalized groups—in the manner seen in Canada, Denmark, New Zealand, Germany, and supported by the International Covenant on Civil and Political Rights (ICCPR). These scholars emphasize the harm that is incurred by hate speech. Hate speech that intentionally provokes fear, for example, limits the freedom of individuals who are the target of such speech, especially when a history of violence gives good reason to be fearful (Lawrence 1993, Matsuda 1993, Waldron 2012, Delgado and Stefancic 2017). Legal restrictions on speech that leads to such intimidation, Waldron argues, serve as a public good that benefits the entire social order.

Scholars in this tradition are well-aware of liberal arguments favoring speech freedoms, and related constitutional principles such as equal protection before the law. But such arguments ring hollow in the face of historical experience in which constitutional guarantees were either formally or informally denied to minority groups (Matsuda 1993, Delgado and Stefancic 2017). Conservative appeals to standards of “equal treatment” in the contemporary context, they argue, freeze in place the highly unequal status quo. As Fish (1994: 91) argues,

the equality David Duke champions is designed to perpetuate the *in*equalities produced by a history or repression and exclusion; by refusing to take into account what has happened in the past, the rhetoric of “equality for everyone” assures that the privileges of the few will be continued into the future, and, best of all, this policy is able to dress itself in the vocabulary of moral purity. It’s like alchemy or magic: now you see white supremacy, but, presto chango, it is given a new description, and now you see “equality for everyone” with no change whatsoever in the practice or the outcome.

Fish (1994) goes further to say that First Amendment principles are an empty abstraction. Whenever we mark the line between protected and regulated speech, Fish argues, we are engaged in a political act. There is no escaping politics when it comes to speech. Therefore, it is better, in Fish’s view, to give up the fiction that one can be neutral (i.e., non-ideological) in establishing the boundaries around speech that is protected and speech that requires regulation. And in giving up that fiction, we can be *mindfully* ideological and political in championing specific values through specific (non-neutral) legal intervention aimed at advancing the ideals of democratic society, in which people are free from speech that intends to exclude some groups from that society.

Arguments challenging the hardline stance favoring First Amendment freedoms have gained traction in recent years, particularly in the context of the academy (Lawrence 1993). A series of racially charged events, related protests, and the resignation of the president and chancellor at University of Missouri in the fall of 2015 represented a watershed moment.^1^ In the years since, extreme-Right backlash, increased frequency of campus events featuring incendiary speakers such as Richard Spencer and Milo Yiannopoulos, and student protests of more mainstream academic speakers have heightened sensitivities and presented new challenges to liberal defenses of free speech principles on campus. If discriminatory behavior toward a protected class is deemed out-of-bounds, why not discriminatory speech? (Shahvisi 2018, Muldoon 2018).

Further, Derald Wing Sue (2010) observes that speech need not be overtly racist, homophobic, or sexist to be problematic. Sue argues that less-overt forms of discriminatory speech—what he identifies as microaggressions—can have deleterious effects on teaching and learning. According to this view, educators have a responsibility to become attuned to the ways they may be unintentionally introducing bias into the classroom through an ill-considered choice of words or expression. Arguments focused on unintentional or implicit bias in the academy, along with regulatory guidance such as Title IX oversight and “Dear Colleague” letters from the Department of Education, have led to the growth of bias reporting systems on campus, in which students and other members of the community can report (often anonymously) when they experience bias in the classroom, microaggressions, or discrimination.

The argument favoring systems of this kind is that it alerts professors to the impact of their language or behavior, allowing them to course-correct and create a more inclusive learning environment.^2^ Noting that good teachers take students’ likely reactions into account and that students cannot learn if they are triggered, Kessler (2018) advocates for tools like trigger warnings as a reasonable accommodation—much like accommodations for students with learning disabilities. He argues further that colleges and universities hire professionals on the student affairs staff who can provide administrative oversite when such accommodations are needed, just as they do when it comes to students with learning disabilities.

Some have also called for the banning of speech that is clearly false. Conly (2018) points to incendiary campus speaker Richard Spencer when she argues that it is reasonable for institutions of higher learning to prohibit speech that does not meet the bar of academic credibility. Conly points to the fact that Spencer and speakers of his ilk would never be invited to speak inside a classroom environment, and thus, are not worthy of any campus invitation. To be worthy of academic discussion, Conly argues, the views being presented must be plausible. “[S]ome speech,” Conly (300) observes, “simply is not educational: it’s false, obviously false, and too obviously false to serve as a useful focus for intellectual criticism. The limited time students spend in educational institutions should be geared to teaching them as much as we can of what is both important and true.” Conly observes that Spencer’s views do not pass the plausibility test. Conly (2018: 309) concludes, “Educational institutions exist to aid in enlightenment. […] Campuses are different from the public street precisely because they have this mission. Colleges and universities should, then, be a haven from nonsense.” Lending support to this view, Kessler (2018) rejects Mill’s argument that false speech has value, at least in the context of universities, as universities have an interest in sorting true from false ideas and conveying truth to students.^3^

Collectively, the literature on this side of the debate argues that higher education fails to fulfill its mission if it does not provide a hospitable learning environment. The solution, according to this view, is to prohibit demeaning or derogatory speech that intimidates or discriminates against marginalized groups, and to deny a platform to those who perpetuate false ideas that compromise the learning environment. Though little attention is given to the specifics of how such prohibitions are to be administered, university-wide policies banning such speech seems to be the assumed standard, with the accompanying presumption that the university administration or a faculty committee of some kind is the appropriate body to enforce that standard.

## The Bottom-Up Nature of Informal Speech Regulation

In the current debate about campus speech, both sides tend to assume that free speech, i.e., speech that enjoys First Amendment protections, is unregulated once it has passed the First Amendment-protected speech threshold.^4^ Some conservative campus organizations and activist groups argue that this is exactly as it should be. As discussed in the preceding section, others argue that institutions of higher learning are justified in imposing regulations that disallow inappropriate speech that undermines the learning environment. Both sides, however, tend to ignore the many ways that speech is regulated by informal, bottom-up, disciplinary forces. In other words, there is a vast “middle ground” of privately regulated speech—speech that enjoys First Amendment protection, but nonetheless is constrained by bottom-up disciplinary forces.^5^

### The Regulatory Power of Enculturation

Included in the middle ground of privately regulated speech are the multiple private and social spaces in which we are nudged, taught, persuaded, encouraged, cajoled, told, scolded, or shamed to say one thing and not another. Consider, for example, speech prohibitions parents typically place on their children. Households with small children are not governed by First Amendment principles but are instead coaching environments in which parents course-correct when a child’s speech and expression are out-of-bounds. Often without having to give the matter conscious thought, the parent sends a corrective signal if an utterance is insensitive, too harsh in tone, or made at the wrong moment. If the parent has done his or her job well, over time, the child internalizes a complex and nuanced set of language customs and protocols that allow the child to successfully navigate the world beyond the home, knowing more or less, what can be said, when it can be said, and how to say it.

As Adam Smith ([1759] 1985) observed in *The Theory of Moral Sentiments,* parents are just the beginning of the process of coaching a child toward expertise in navigating the social world. Indeed, modern psychologists concur with Smith that peers play a critical role in a child’s moral and emotional development (Ladd 2005). Peers check behavior, speech, and expressive acts that fall outside commonly accepted boundaries. Here again, the rules that govern appropriateness of what can be said adjust in nuanced ways depending on context. For example, what can be said among close friends, to newcomers, in classrooms, to one another’s parents and siblings, etc., changes, depending on age, familiarity, local cultural norms, and the subject at hand. (As will be discussed below, it is this context-dependent dimension of how human beings learn to adjust language rules to different circumstances that make language resistant to top-down control.)

As we grow beyond the context of our childhood home and friends, we gain entry into more formal language environments. Standards of appropriate conduct in school, through mechanisms that are both formal and informal, establish what is considered inbounds and out-of-bounds in early learning environments. Professional spaces are similarly regulated. Students, faculty, and staff arrive on campus, therefore, having already been enculturated within specific speech communities, already in possession of built-in governors regulating what they say, when and how they say it, and to whom they say it.

In other words, there is no such thing as unregulated speech.^6^ Language communities are highly regulated communities. Unlike regulatory regimes that craft and pass down inflexible rules from the top-down, however, language is regulated through a decentralized bottom-up process, with countless nodal points of localized authority (e.g., parents, teachers, coaches, siblings, peers), each experimenting with and deploying rules intended to serve their purposes.

That we arrive on campus with a built-in governor is not itself a remedy to the challenges we face with on-campus speech, but it is a starting point. The university environment offers another level of bottom-up regulation in the form of academic gatekeeping.

### The Regulating Power of Academic Gatekeeping

In an essay describing the positive and negative consequences of banning controversial speech, Elizabeth Barnes^7^ writes,

I think that there are some ideas that shouldn’t be engaged with. If a fellow philosopher tells me that they have an argument for the moral goodness of rape, I quite simply don’t want to hear it. I won’t go to the talk, I won’t invite that person to my conference, I won’t read that paper. I don’t think the argument deserves attention. Saying why, though, is tricky…

Barnes goes on to discuss the rationale behind “the why.” Academics have to weigh the costs and benefits of engagement. In Barnes’ estimation, “A pro-rape argument isn’t an important ‘option on the table’ in debates about sexual ethics […]” On the other hand, Barnes does consider Peter Singer’s arguments favoring euthanasia of infants with certain severe disabilities worth engaging, because, Barnes concludes, Singer is saying explicitly what many others are thinking—that everyone would be better off if the lives of some infants were terminated.^8^ Despite her reservations about Singer’s conclusions, Barnes acknowledges that it is her responsibility, particularly as an academic with a great deal of privilege, to engage with difficult ideas that make her uncomfortable.

In thinking through when it is appropriate to engage an argument, and when it is better to not engage, Barnes calls attention to a ubiquitous and necessary practice—one of intellectual gatekeeping. In their choice of readings, topics for discussion, invitations of guest speakers, and scholars to engage in their scholarship, academics select content into and out of the classroom, public spaces within the university, and scholarly research. In a world of scarce time, financial resources, and attention bandwidth, there is no escaping such decisions. And in their role as scholars and teachers, academics are professionally obliged to think seriously about these tradeoffs, and to do their best to make sound curatorial judgments. Such curatorial judgment is a regulatory process.

One lesson we might take away from this is that since intellectual gatekeeping is something all academics do, all the time, it might make sense to deploy that ubiquitous gatekeeping function across the institution as a whole. In keeping with other advocates for institution-wide bans on hate speech, this is the argument Conly (2018) makes with regard to speech that is obviously and clearly false, such as a policy that would refuse a platform to Holocaust deniers. By imposing a ban on speech that is clearly false and/or has the potential to cause great harm, the logic goes, the institution simply enforces standards that academics impose on their work on a daily basis.

Below, I argue against this position. That said, framing the issue as Conly does draws attention to the right questions. The question is not *whether* to regulate academic speech. As noted above, from child-rearing to peer-group interaction, to professorial discretion, speech is fundamentally embedded within a regulatory process. Rather, the central questions are *how* academic speech should be regulated. By what criteria? And under whose authority? By framing campus speech questions in these terms, we have reason to explore more deeply the regulatory power of this ubiquitous practice of decentralized bottom-up curatorial decision-making and compare the pattern of outcomes that emerges from such a practice to the top-down regulatory approach implied in calls for campus-wide bans on hate speech and speech that is clearly false.

## A Bottom-Up Approach to Campus Speech Governance

Before discussing the elements of a bottom-up approach to speech governance and how it compares to a top-down approach, it is important to identify standards of performance and outcomes we would want to see, regardless of which approach is taken. Ideally, a system of governance avoids strong tendencies toward the abuse of power and authority, e.g., using rules and procedures to punish political or ideological rivals. Further, a well-functioning system of governance generates a high degree of coordination and minimizes conflict, i.e., people can get on with the business at hand at relatively low cost. Finally, a system of governance should generate outcomes that align with the goals of the institution. In the case of speech governance, we would want to see patterns of decision-making that foster a climate of intellectual integrity and an environment conducive to teaching and learning.

An institution-wide ban on hate speech or speech that is clearly false represents the ideal-typical top-down approach to speech governance. Such a ban carries with it the burden of identifying criteria for speech that is to be considered within bounds and speech that will be considered out-of-bounds. Given the complex and nuanced nature of language, however, perfect specificity is impossible. Some person or persons—an administrator or speech oversight committee—will have to be named to adjudicate close calls and identify appropriate sanctions when necessary.

The alternative I propose here, again in ideal-typical terms, is a bottom-up approach to speech governance that rests on there being many decentralized sources of localized authority and curatorial control, rather than a single centralized source of regulatory power. The bottom-up approach to speech governance rests upon two basic principles. First, baseline protections of academic freedom and due process are guaranteed. Second, in practice, a default respect for curatorial decisions made at the local level operates, with higher levels of authority stepping in, in measured and graduated fashion, and only when necessary.

These basic principles can be found in some form at most American institutions of higher learning. The first serves as a “constitutional framework” that appropriately limits the powers of the majority and administrative authorities.^9^ Without guarantees of academic freedom and due process, any form of governance is in danger of degrading into tyranny of either the majority or a concentrated political authority. A majority vote of the Faculty Senate, for example—even a unanimous vote—cannot abridge the academic freedom of the individual, nor deny due process, if such dangers are to be avoided. In a constrained democratic process, in other words, some powers are simply off the table. While these “constitutional guarantees” have been challenged in recent years, most colleges and universities recognize the value of these baseline rules of the game and seek to adhere to them in practice.^10^

**Fig. 1:**
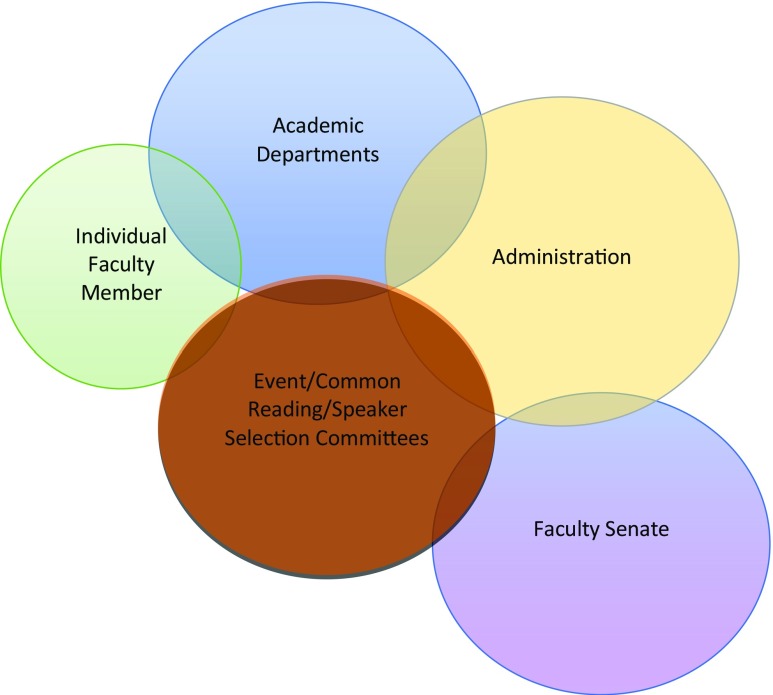
A polycentric bottom-up model of speech governance

The second basic principle—default respect for decisions made at the local level—will also sound familiar to most academics, as it is the underlying principle at work within most models of faculty governance. Building off of the work of Elinor and Vincent Ostrom and the Bloomington School of political-economy, Aligica and Chamlee-Wright (2015) argue that faculty governance constitutes a “polycentric order” in which multiple overlapping spheres of authority comprise a decentralized, bottom-up system of governance (Ostrom 1990, McGinnis 1999). Figure 1 represents this process in the context of speech governance.

Consider the gatekeeping function faculty play in selecting topics, readings, and guest speakers in their own courses. In Fig. 1, this presumed authority is represented by the area of the “Individual Faculty Member” sphere that does not intersect with other spheres. This authority is non-arbitrary. It is widely recognized within the academy that faculty possess the disciplinary expertise and local knowledge of the student population needed to render informed judgment. Further, given the importance of reputation in the higher education industry, faculty have a strong incentive to consider their choices carefully and to exercise sound judgment.^11^ The authority to invite outside speakers often extends to public spaces beyond the classroom, time and space permitting. Here again, the expertise of the individual faculty member, coupled with a desire to uphold his or her professional reputation among colleagues, is generally a sufficient level of regulatory control.

Large-scale events, especially those requiring significant funding, are often issued under the auspices of departmental sponsorship, or as part of a funded speaker or event series. In Fig. 1, the expectation of shared decision making is represented by the overlap between the Individual Faculty Member sphere and other, next-level-up spheres of authority (i.e., academic departments and selection committees charged with overseeing an event or speaker series, or common reading). Under such circumstances, a small group of decision makers may operate informally or they may deploy an agreed upon framework for determining which speakers, exhibitions, performances, or events are selected for invitation.

Speakers receiving honorary degrees and a high-profile platform, such as the graduation stage, are often vetted by a faculty committee, which makes a recommendation to the university administration for approval. At some institutions, such selections, especially where an honorary degree is concerned, are subject to a vote of the Faculty Senate. (See relevant areas of overlap in Fig. 1.) In other words, when the stakes are relatively low, and the consequences of a good or bad decision are relatively localized, it is appropriate to allow the individual faculty member to exercise his or her gatekeeping authority on their own. As the stakes grow, so too does the degree of shared governance, and the formality of the process.

From this bottom-up regulatory process, an order emerges. Individual faculty, academic departments, speaker series selection committees, and so on, operate as a decentralized curatorial system that tends to weed out speech that compromises intellectual integrity or degrades the learning environment. The argument here is not that every topic or text a faculty member selects, or that every campus speaker extended an invitation through this process avoids controversy. (Given the good that can come of intellectual controversy, this should not be the goal.) Nor is it my argument that *only* the most highly-regarded texts and *only* speakers of an unimpeachable reputation find their way onto campus. (Again, such a standard would be too conservative in that it would not allow for any intellectual risk taking.) The claim is, rather, that the system of overlapping curatorial control we find in operation at most campuses generates a pattern of good outcomes most of the time. Most of the time, selected topics and texts, invited speakers, and the discussions they generate meet standards of academic credibility, are productive of learning, and avoid the most pressing concerns raised by advocates of campus-wide bans on problematic speech.^12^

## Political and Epistemological Properties of Top-Down and Bottom-Up Governance

In what follows, I consider how a top-down structure of speech governance works in practice, so as to compare its systemic properties to those emerging from a bottom-up speech governance process. For the sake of discussion and analytical clarity only, I set aside the question of whether the rules of operation in a top-down process could withstand a First Amendment challenge at a public university. Aside from First Amendment concerns, a top-down approach to speech governance generates two additional categories of concern: political economy problems, and problems that are epistemological in nature. As I argue below, a bottom-up approach, on the other hand, possesses emergent properties that guard against these hazards.

### Political-Economy Implications of Top-Down Speech Governance

As noted above, language is complicated in ways that render it resistant to specificity in rule making. Taboo words and imagery, for example, pose a particular challenge. Consider a theatrical production depicting characters in blackface. What should the posture of the academic community be toward such a production? The answer will depend on how the imagery is used. A production that replicates the racist overtones of the nineteenth century minstrel show will be judged differently from a production that recalls the potent image of players in blackface ironically. (Consider, for example, Robert Earl Price’s theatrical production *All Blues.*) It is likely impossible, in other words, to establish a rule, a priori*,* that would cover all contingencies appropriately.

Given the difficulty of specifying rules that cover all contingencies, the top-down approach will necessarily involve a great deal of discretionary judgment by the person or persons overseeing adjudication and enforcement of the speech restrictions. This raises then the crucial question of *who* it is that will exercise that discretionary authority. A faculty committee charged with overseeing the speech restriction may be the answer. Again, considering the subtleties of language—that the same utterance may be deemed hate speech (or not) depending upon context, who is doing the speaking, their presumed intent, who is listening, and so on—such a committee would possess considerable discretionary power over students and their colleagues.

As F.A. Hayek (1944) might have warned, a committee with such concentrated power might very well attract faculty who are bent on some form of ideological purity test. It doesn’t matter whether the ideological agenda comes from the conservative or progressive end of the spectrum. The danger is present either way. More broadly, given the significant discretionary authority that such a committee would enjoy, we have reason to be concerned that the oversight committee has the potential to become a speech tribunal to which “misbehaving” faculty will have to submit if they say anything controversial in or beyond the classroom. Recognizing these dangers, the impulse may be to place this authority in administrative hands. But handing this level of discretionary power to a dean, provost, or president raises similar concerns, as such discretion would give that administrator the power to override academic freedom, as well as provide scope to punish adversaries on the faculty if the administrator were so inclined.

Some advocates of a top-down approach concede that “hate speech” is too broad a category to police, and suggest instead a narrower prohibition, on for example, speech that is clearly false—ideas that clearly have no standing within the academic community (Conly 2018). Speech would be banned only in the case where the conclusions drawn are obviously wrong—in the case of Holocaust deniers, for example—with the implication that no academic value can come from further debate on the issue. Ideas that are clearly *not* settled, Conly assures, would be open for debate and discussion. But the problem with the “clearly false” criterion is that there will always be cases in which some will insist that the proposition being advanced is “clearly false” while others will say that the speech in question has not crossed that line. The magnitude and causes of global warming, for example, and propositions regarding “racial fluidity” as a meaningful concept come to mind. Cases like these will require that we vest in some entity (an administrator or a speech oversight committee) discretionary power that raises the same concerns discussed above.

When considered from the perspective of a “bottom-up” approach, on the other hand, speech that is clearly false is largely addressed by the fact that faculty have already curated out, as a matter of basic professional integrity, those speakers and points of view that hold no sway within the academic community. Thus, a university ban on speech that is clearly false is unnecessary, and only serves to muddy the waters when a faculty member assigns a text or speech he or she knows to be false as an object of scrutiny, e.g., to engage in a sociological analysis of Holocaust deniers by scrutinizing their arguments first-hand.

More generally, a bottom-up approach to speech governance excludes the worst, most-implausible ideas most of the time, while still leaving the discursive space contestable, with respect to who holds power over that space. Whereas a top-down approach allows for only one gatekeeper with concentrated power, a bottom-up approach fosters a system of decentralized gatekeeping, each with only a limited sphere of localized authority. In a decentralized system of localized authority there is no source point of concentrated power over which ideological or political rivals compete. In other words, while there may still exist individuals with a desire to engage in ideological or political control and manipulation of their colleagues, they do not have access to a mechanism to do so. The best they can do is exercise curatorial control over their limited sphere of authority, leaving the rest of the discursive space open for intellectual exchange.

### Epistemological Implications of Top-Down Speech Governance

For the sake of argument, let us suppose that we are able to solve the political-economy problems associated with top-down speech governance. Imagine, for example, that we have found the academic equivalents of the *Start Trek Next Generation* character Salia from the episode “The Dauphin” —raised to be just, pure-of-heart, and a neutral sovereign over warring factions—to serve lifetime appointments on the campus speech oversight committee. Even with political economy problems set aside, however, another problem arises, this one epistemological in nature.

Whether its task is to determine what speech is “clearly false,” or to adjudicate alleged incidents of hate speech, a speech oversight committee faces a challenge of complex interpretation. For example, a review process to determine whether a proposed guest speaker’s conclusions are clearly false, and therefore not worthy of a university platform, is likely to involve competing claims and specialized knowledge from multiple disciplines. When adjudicating cases of alleged hate speech, the committee must make fine distinctions between utterances that are intentionally aimed at disparaging a particular group, utterances that unintentionally wound or offend, and speech that, though provocative, is in service to an artistic or pedagogical purpose. Making such distinctions is essential if the committee is to assign the appropriate sanction, or in the case of the latter scenario, no sanction.^13^ Given the complexity of the interpretive task, therefore, it is important to understand what categories of knowledge such an oversight authority will and will not have at its disposal.

In carrying out its charge, the speech oversight committee faces a knowledge deficit in at least three respects. First, such a committee will likely lack the disciplinary expertise needed for any specific speech situation. Second, committee members will not possess relevant local knowledge cultivated by boots-on-the-ground decision makers. Third, to the extent that top-down decision-making replaces the decentralized curatorial process of speech governance, the committee is cut off from the systemic discovery that otherwise unfolds from the bottom-up.

To the first point, the speech oversight committee will be limited in the disciplinary expertise it is able to bring to its governance task. Every member of the committee may agree, for example, that a particular argument is abhorrent, but as Miller (2018) observes, an abhorrent argument may still have a legitimate claim of plausibility. Disciplinary expertise is often necessary to sort abhorrent claims that lack plausibility from claims that may be plausible, even if they are widely considered to be abhorrent. Similarly, provocative material on taboo subjects that may seem obviously out-of-bounds to, say, the typical natural scientist, may be commonplace in courses like *Sociology of Deviance*, *Cultural Anthropology,* or *Forbidden Literatures.*

In the context of a bottom-up approach, on the other hand, decision makers at the local level bring professional expertise and experience to bear in their judgments of which topics, texts, arguments, and speakers are appropriate to introduce to their classroom and the campus community. If those decision makers require guidance or coaching, departmental colleagues with relevant disciplinary expertise are close-at-hand to offer that support. In other words, relative to a top-down authority, a bottom-up approach to speech governance makes better use of disciplinary-based knowledge and expertise, because the local decision-maker has direct or proximate access to that knowledge. The top-down authority is much farther removed from that knowledge base.

Further, in addition to a deficit in disciplinary-based expertise, the speech oversight committee lacks what Hayek ([1948] 1984) referred to as “local knowledge.” In the context of the classroom, local knowledge includes awareness of small but relevant details that foster learning of a particular concept, knowledge of how a particular topic needs to be framed or presented to overcome student resistance, on-the-spot knowledge that a particular group of students is simply missing the point of the presentation, and informed hunches about how to adjust one’s approach in the face of that challenge. Local knowledge is the sort of knowledge decision makers typically acquire by being embedded in the decision-making context day in and day out. In their role as localized gatekeepers, faculty come to know, for example, the subtle distinctions between speech that is merely provocative and speech that is productive of learning (even if it is also provocative). They learn how to manage the negative impact some speech may have on the listener by framing it carefully, and by following up sensitively, with open or private discussion with students who find the speech disturbing.

On the other hand, because it is removed from the context in which boots-on-the-ground knowledge is cultivated, the speech oversight committee does not possess this knowledge. This knowledge gap is problematic if the oversight committee’s intent is to supersede the better-informed judgment made at the local level. One might object that faculty who frequently exercise poor judgment need some sort of course-correction. Indeed. But the relevant question is not *whether* a poorly performing faculty member needs course-correction; the question is *who* should provide it. A bottom-up approach to speech governance suggests that rather than a relatively distant speech oversight committee, departmental colleagues and a department chair are generally better positioned to offer necessary guidance, as they are more likely to have access to the local knowledge that will inform the coaching process.

The most significant knowledge gap a top-down approach to speech governance presents is inherent within the top-down governance process itself. The more active the top-down authority is in overriding decisions made at the local level, the less-inclined local decision makers will be to exercise independent judgment. If a faculty member assumes that there is a high likelihood of top-down intervention, the default will increasingly be, “better ask permission first,” rendering decision-making costlier, which in turn reduces the frequency of experimentation and slows the pace of learning that comes from experimentation. The more top-down decision-making replaces the decentralized system of curatorial gatekeeping, in other words, the more it erodes the discovery that is inherent within the iterative bottom-up process.

As noted above, because it allows for competing judgments from many decision-makers, each with only localized authority, a bottom-up approach leaves the intellectual/discursive space on campus a contestable space. This contestability brings with it epistemological advantages (Hayek 1978). Because decision makers do not have to seek and secure permission to exercise their local authority, the costs of experimentation and learning are relatively low, and the iterations of experimentation and learning will be relatively high. Further, contestability in the discursive space affords greater scope for the system to compensate for error. For example, suppose that a given member of the faculty has been unfairly narrow in her curatorial decision making. Such narrowness nonetheless leaves the intellectual space of the university open for competing points of view to enter, with hundreds to thousands of other faculty and courses giving worthy ideas a shot at a fair hearing.

The claim here is not that faculty always make wise decisions. On the contrary, we can be certain that well-intended academics frequently fail to hit the mark. But this fact only underscores the importance of leaving the discursive space contestable, since there is a good chance that any single person or committee will get the judgment wrong, even if they are well-intended. It is therefore all the more important that no single person has concentrated power over the process of speech governance.^14^ What we should be seeking is a system that takes advantage of the benefits of informed judgment, but limits the damage done when we invariably get it wrong. If individual curators get it wrong, the harm is localized, and we leave open the possibility for correction. On the other hand, if an administrator or speech oversight committee with centralized power gets it wrong, the impact is campus-wide, and there is limited scope for further experimentation, learning, and course-correction.

## Open Questions and Lingering Concerns

The argument made here raises questions that deserve further consideration. For example, the foregoing analysis focuses solely on the curatorial authority faculty exercise and does not address the question of whether students should have similar gatekeeping authority. This is an important question, as many of the campus speakers generating the most controversy have been invited by student groups sanctioned to exercise this authority. Viewed through the lens of the bottom-up paradigm, students represent another layer of the governance process that is in some ways similar to the localized authority exercised by faculty. But in other important respects, student groups are different than faculty, in that they lack disciplinary expertise and the long-term incentive to maintain their reputation. Therefore, more consideration needs to be given for how a bottom-up approach informs this debate.

Relatedly, given the problems associated with incendiary “shock” speakers who have gained entry onto campus, we should be open to discuss the discursive costs and benefits, and potential legal pitfalls, associated with a policy that required sign-off from a current member of the faculty before any speaker is permitted access to a campus venue. Again, further consideration from a bottom-up perspective is warranted.

The bottom-up approach to speech governance described here also raises the question of whether faculty have a responsibility to not only curate *content* that leads to productive learning, but also a responsibility to cultivate discursive norms that foster productive learning, and if so, what those norms are. For example, do interlocutors have a responsibility to be sensitive to the emotional response their argument is likely to inspire? Do interlocutors have a responsibility to be resilient upon hearing speech that they find disturbing? If so, what are the limits on these expectations, and how do we cultivate these discursive habits? Relatedly, what are the lines of demarcation between a bottom-up disciplinary force that productively abrades the sharp edges of speech, and bottom-up disciplinary force that unproductively silences diverse points of view?

Perhaps most pressing are concerns related to self-censorship and how a bottom-up approach to speech governance might address those concerns. Guarantees of academic freedom and due process help to ensure that the vocal member of the academic community is protected against “hard” forms of tyranny, e.g., termination for advancing a particular point of view. But soft forms of tyranny are also present in the academy. Tactics range from mild social distancing, to ostracism, to over-zealous policing of procedural compliance, to calling out on social media, to accusations of professional impropriety (Kipnis 2017). The life of the mind is intimately connected to the community of scholars with whom we associate. To be cut off from or threatened by that community can be emotionally and professionally devastating. The response to such tactics, or the threat that such tactics might be used is often self-censorship (Bromwich 2016). Self-censorship is likely a problem across the board, but it is worth asking the question whether a bottom-up system of governance, coupled with robust discursive norms championing the open exchange of ideas might improve the current state of higher education.

Finally, the foregoing argument makes much of the tendency for a bottom-up approach to leave the discursive space contestable. It is this contestability that renders the marketplace of ideas a driving force of system-wide discovery, even when individuals make errors in judgment. But if the local cultural environment is highly constrained by the soft forms of tyranny mentioned above, the system of bottom-up governance may be prone to bias favoring the dominant point of view. Therefore, bottom-up governance is no panacea for concerns related to viewpoint diversity and corrosive academic cultures that shut down rather than open up the marketplace of ideas.

The open questions and lingering concerns briefly described here are the subject of future inquiry.

## Conclusion

I have argued that through a bottom-up process of speech governance it is possible to attend to challenges around campus speech, such as speech that lacks academic credibility and speech that disparages members of oppressed groups, without undermining the principles of free speech and academic freedom.

The proposed bottom-up solution relies on operating principles familiar within the academy, namely, guarantees of academic freedom and due process, and respect for curatorial discretion made at the local level. Guarantees of academic freedom and due process serve as the safeguard against tyranny-of-the-majority-type problems by placing appropriate limits on what is “on the table for consideration” in the governance process. Default respect for decisions made at the local level ensures that individuals can exercise their best judgment in curating in appropriate speech, and curating out inappropriate speech, within their limited sphere of localized authority.

With these principles in place, we avoid the political economy problems associated with highly concentrated source points of power, namely, the likelihood that such power will attract those willing to erode the principle of academic freedom and eager to impose ideological control over their colleagues. Further, the bottom-up approach allows faculty, as individual caretakers of their classroom, as members of academic departments and small groups charged with the selection of campus speakers and texts to make frequent use of expertise and local knowledge they possess without incurring the costs of seeking and securing permission from higher levels of authority. This ease of informed decision-making fosters higher frequency in experimentation, allowing faculty to learn, by trial and error, how to make better curatorial decisions. The fact that the relevant spheres of decision-making authority are limited means that the discursive space is left open to competing points of view and challenge. Though the bottom-up approach to speech governance is not a panacea, relative to a top-down approach, the decentralized system of decision-making authority is more likely to lead to a pattern of outcomes that align with institutional goals, such as a climate marked by intellectual integrity and an environment conducive to teaching and learning.

## Footnotes

1 See Alia Wong and Adrienne Green, “Campus Politics: A Cheat Sheet.” *The Atlantic*, April 4, 2016. https://www.theatlantic.com/education/archive/2016/04/campus-protest-roundup/417570/

2 Bias reporting systems have also drawn criticism for curtailing academic freedom and failing to afford faculty due process. See Nadia Dreed, “Amid Concerns About Stifling Speech, Colleges Take a New Look at Bias-Response Teams,” *Chronicle for Higher Education,* September 28, 2016. https://www.chronicle.com/article/Amid-Concerns-About-Stifling/237918

3 Mill’s (1859 [2002]) argument has two parts. First, epistemological humility: we need to be tolerant of speech we believe to be false because it may turn out that we are wrong. Second, positive effects of abrasion: arguing against bad ideas keeps us intellectually sharp and gives animating force to good ideas.

4 While the first amendment protects hate speech, it does not offer such protection to speech that is directly threatening or defamatory. Nor does it protect speech or forms of expression that can result in immediate and significant harm to others, such as shouting “fire” in a crowded theatre when no fire exists.

5 When we hear the word “regulation,” we often assume that government or some other top-down authority is the source of regulatory discipline. The work of Elinor and Vincent Ostrom and their colleagues at the Workshop in Political Theory and Policy Analysis at Indiana University, Bloomington challenges this assumption. See McGinnis (1999) for discussions of “polycentric governance”—structures of governance in which decision-making authority is decentralized and governing discipline emerges in a bottom-up fashion.

6 In his book (and essay of the same title) *There’s No Such Thing as Free Speech*, Stanley Fish (1994) argues that speech is never “free” because the formal rules governing speech are always reflective of the ideology of those who wield the power to define those rules. In arguing that there is no such thing as unregulated speech, I am making a different point—that there are multiple source points of regulation, each with varying degrees of power and authority.

7 See Elizabeth Barnes, “Arguments that Harm and Why We Need them,” *The Chronicle of Higher Education*, February 18, 2018.

8 In this regard, Barnes is making an argument that is similar to Miller’s (2018) conclusion that good can come from listening to arguments with abhorrent conclusions if the interlocutor is a “sincere agent.” As Barnes describes him, Singer is a sincere agent, even though she disagrees vehemently with his conclusion.

9 See Buchanan (2000) on the importance of constitutional constraints.

10 The Halloween costume controversy at Yale, the Day of Absence controversy at Evergreen State University, the Eudaimonia Institute controversy at Wake Forest University, and personnel actions taken without due process at University of Nebraska, Lincoln serve as cautionary tales of what can happen when student opinion (in the case of Yale and Evergreen) or a faculty senate vote (in the case of Wake Forest) or a state legislature (in the case of University of Nebraska) usurps academic freedom and due process.

11 It is worth noting, however, that no professor is an island. If an individual faculty member fails to exercise good judgment, the department chair may intervene with strong counsel. In such a case, the burden is on the chair to ensure that the intervention is in the name of maintaining professional standards and does not violate the faculty member’s guarantee of academic freedom.

12 When an institution hosts non-academic events, a similar process is often deployed by members of the relevant administrative staff.

13 See Johnson (2018) on how comedic speech problematizes attempts to impose top-down solutions to speech governance along these lines.

14 See Cohen (2014) for a discussion of the local and society-wide effects, in terms of truth acquisition, of suppressing vs. tolerating false speech.
